# The SOS Response Activation and the Risk of Antibiotic Resistance Enhancement in *Proteus* spp. Strains Exposed to Subinhibitory Concentrations of Ciprofloxacin

**DOI:** 10.3390/ijms26010119

**Published:** 2024-12-26

**Authors:** Agnieszka Zabłotni, Marek Schmidt, Małgorzata Siwińska

**Affiliations:** Department of Biology of Bacteria, Institute of Microbiology, Biotechnology and Immunology, Faculty of Biology and Environmental Protection, University of Lodz, 90-237 Lodz, Poland; marek.schmidt@tome.com.pl (M.S.); malgorzata.siwinska@biol.uni.lodz.pl (M.S.)

**Keywords:** *Proteus*, subinhibitory concentrations of antibiotics, ciprofloxacin, antibiotic resistance, SOS response

## Abstract

The widespread and inappropriate use of antibiotics, for therapeutic and prophylactic purposes, has contributed to a global crisis of rapidly increasing antimicrobial resistance of microorganisms. This resistance is often associated with elevated mutagenesis induced by the presence of antibiotics. Additionally, subinhibitory concentrations of antibiotics can trigger stress responses in bacteria, further exacerbating this problem. In the present study, we investigated the effect of low doses of ciprofloxacin on the induction of the SOS response and the subsequent development of antibiotic resistance in *Proteus* spp. strains. Our findings revealed an increase in mutation frequencies within the studied strains, accompanied by a significant upregulation of *recA* expression. These observations were consistent across experiments involving two subinhibitory concentrations of ciprofloxacin. To establish mutation frequencies and assess gene expression changes, we utilized the Rif^S^-to-Rif^R^ forward mutagenesis assay and RT-qPCR analysis, respectively. Furthermore, employing the microdilution method, we demonstrated that these changes could promote cross-resistance to multiple classes of antibiotics in *Proteus* spp. clinical strains. This, combined with the recurrent nature of *Proteus*-associated infections, poses a substantial risk of therapeutic failure. In conclusion, exposure to low doses of ciprofloxacin can significantly impact the susceptibility of *Proteus* bacilli, not only reducing their sensitivity to ciprofloxacin itself but also fostering resistance to other antibiotic classes. These findings underscore the importance of cautious antibiotic use and highlight the potential consequences of subinhibitory antibiotic exposure in clinical and environmental settings.

## 1. Introduction

Antibiotics—the main tool for combating infectious diseases—still remain one of the most important achievements of medicine, allowing for us to save the lives of millions of people around the world each year. This position remains unchanged, even if we take into account that drug resistance has been increasing steadily for 70 years, since the introduction of the first antibiotic in medical use. Our knowledge about different mechanisms of bacterial drug resistance has evolved from a classic view (selection of already pre-existing resistant microorganisms in contact with antibiotics leading to their dominance in population) to the current understanding this phenomenon, which also takes into account horizontal gene transfer or recombination [1,2,3].

Many antibiotics in bactericidal or bacteriostatic concentrations can cause bacterial DNA damage directly, like quinolones, or indirectly through induction of endogenous reactive oxygen species (ROS) formation. This last mechanism is common for several classes of antibiotics and results not only in DNA damage but also affects other important cell structures including proteins or lipids [2,4,5]. Stress associated with damage and the appearance of single-stranded DNA (ssDNA) leads to the bacterial SOS response activation through derepression of SOS regulon genes and transcription of inter alia three DNA polymerases: II, IV, and V. During the DNA repair process, these error-prone polymerases introduce mutations into the bacterial genome, resulting in the appearance of new adaptive phenotypes [6,7,8,9].

It was clearly demonstrated that SOS response could also be activated when antibiotics are present in much lower concentrations than those used in medical treatment to eradicate microorganisms (subinhibitory concentrations, SICs). In this case, undirected modifications of a wide range of genes (even genes for carbohydrate metabolism or protein synthesis) are noticed. It was also confirmed that antibiotics at such concentrations may act as signaling molecules, changing the transcription of 5–10% genes in bacterial cells. Changes in transcriptional level do not refer only to the genes that are the target of the antibiotic activity, and therefore the antibiotic-induced modulations at the transcriptional level result in many different phenotypes [10,11,12,13,14,15].

There is strong evidence that the SIC of antibiotics via SOS defense induction may contribute to great changes in the drug susceptibility of bacteria. The occurrence of antibiotic resistance, in particular cross-resistance, induced by low doses of various antibiotics, especially quinolones, has been reported, e.g., in *Pseudomonas aeruginosa* or *Escherichia coli* [2,9,16,17,18,19]. However, there is scarce information about changes induced by SICs of antibiotics in Gram-negative, nonsporulating, rod-shaped bacteria from the genus *Proteus*, belonging to the family *Morganellaceae* [20,21]. These opportunistic bacteria have been identified as etiological agents of meningitis in neonates or infants, pneumonia, bacteremia, endocarditis, brain abscesses, wounds, and bone infection and have also been isolated from feces as a part of natural microbiota. Undoubtedly, the most frequent among *Proteus* infections are urinary tract infections (UTIs), often recurrent, catheter-associated, and complicated [21,22,23,24]. The hallmark of this kind of infection is the formation of urinary stones, which complicate the treatment of the disease since their porous structure is a safe shelter that antibiotics are not able to penetrate [25,26,27]. Therefore, antibiotics used to eliminate *Proteus* spp. bacilli do not reach every part of a stone and, in consequence, indwelling microorganisms are sometimes exposed to SICs of the drugs for a long time. Also, distribution of antibiotics in wounds or feces (the second and third source of isolation of *Proteus* bacilli, respectively) can also be stymied and uneven; thus, microorganisms in these places can be subjected to the action of SICs, too.

Given the limited information on the influence of SICs of antibiotics on *Proteus* spp. bacilli and the widespread use of ciprofloxacin in treating *Proteus*-mediated infections, our study aimed to investigate whether low doses of ciprofloxacin could trigger an SOS response, enhance mutagenesis, and alter antibiotic susceptibility profiles in *Proteus* cells. By exploring the effects of action of SICs of ciprofloxacin, this study aimed to determine whether these conditions can promote genetic changes in *Proteus* spp., potentially influencing their ability to adapt to stressful conditions. Understanding these dynamics is crucial for assessing how SICs might inadvertently contribute to resistance development and treatment challenges.

## 2. Results and Discussion

The role of antibiotics in SICs has been widely described and discussed in the literature in recent years [17,28,29]. They may act as signaling molecules and therefore modulate the transcription level of different bacterial genes, but also, DNA-targeting fluoroquinolones especially can increase the frequency of mutations. The antibiotic-induced modulation of transcription and increasing mutation rates result in the occurrence of new adaptative phenotypes in bacterial populations that can be clinically dangerous [30,31].

It has been shown that the SICs of antibiotics as stress-mediating factors may enhance the expression level of the *recA* gene—a key component and inducer of SOS response. This can result in the increased mutation frequency due to the activation of the genes of so-called DNA repair polymerases, which, in order to sustain replication, introduce no targeted mutations into the genome [32,33,34,35]. Although the influence of SICs of fluoroquinolones on changes in the mutation frequency of some microorganisms has already been described [9,16,36,37], most of the data concern pathogenic or model bacteria. Little is known in relation to opportunistic pathogens—including the subject of this study, *Proteus* spp. bacilli. Opportunistic microorganisms do not cause infections in immunocompetent hosts; however, when the immune response is decreased, they can lead to serious diseases and increased mortality [38,39,40]. In such cases, a change in their phenotype to one more virulent or more drug-resistant—resulting from changes indirectly caused by an increased frequency of mutations due to the action of SICs of antibiotics—may be particularly dangerous.

The eradication of urinary tract infections, including those of *Proteus* spp. etiology, is very often based on the use of fluoroquinolones, especially ciprofloxacin (CIP)—the second-generation antibiotic of this group [41,42]. Taking into account the above and due to the lack of data on the influence of low doses of CIP on the mutation frequency in opportunistic *Proteus* spp. cells, we decided to check it, and to our best knowledge, this is the first report on this subject. The Rif^R^ mutants’ selection assay was used as a method for mutation rate estimation, since this methodology is often recommended for establishing mutation frequency in various bacteria [34,36,43,44,45,46]. Strains were selected to include isolates from three most common sources of isolation: urine—*P. mirabilis* (*P.m*.) 9Bm and *P. vulgaris* (*P.v.*) Bm78 and Sm95; wounds—*P.m.* Br19 and Dr64 and *P.v.* Kr83; and feces—*P.m.* Dk32, Dk45, and Bk100 (see Table 3 in Section 3).

**Figure 1:**
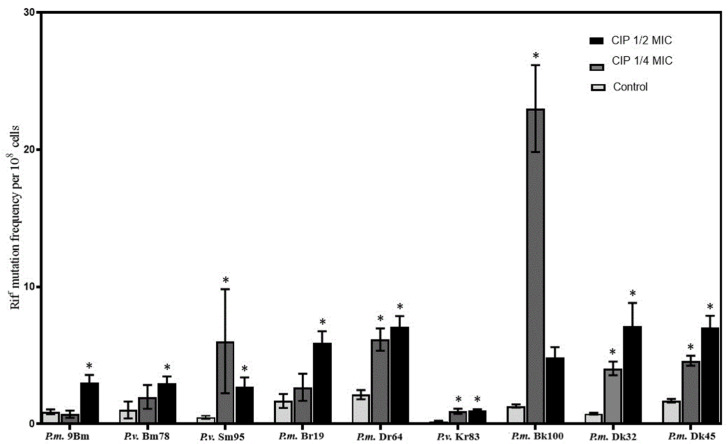
Rifampin resistance mutation frequencies observed after growth in MHB medium with or without ciprofloxacin (in concentrations corresponding to ¼ or ½ MIC). * indicates *p* < 0.05.

The obtained results (Figure 1) showed that a significant increase (*p* < 0.05) in the mutation frequency was noted for the all of the tested strains, and in most cases, it was observed in the presence of ¼ and ½ MIC of CIP (strains: *P.m* Dr64, Dk32, and Dk45; *P.v.* Sm95 and Kr83). The four other strains showed the increase only in the case of one of the tested SIC of CIP—three of them (*P.m*. 9Bm and Br19; *P.v.* Bm78) in a concentration corresponding to the value of ¼ MIC, but *P.m.* Bk100 strain in ½ MIC. This rise in the emergence of rifampin-resistant mutant frequency in the presence of the used concentrations, in relation to the frequency in cells not treated with antibiotic, varied in a fairly wide range from 2- to even 12-fold (in the case of *P.v.* Sm95 strain), most often between 3- and 5-fold. Thus, we have demonstrated that these opportunistic, Gram-negative rods can mutate at a significantly increased rate if antibiotics are present in their growth environment at concentrations lower than those needed for complete eradication. The level of the increase in the mutation frequency is not uniform and appears to be strain-dependent, although it has been found to be similar to the level of growth previously reported for other Gram-positive and Gram-negative bacteria (e.g., five-fold for *Streptococcus pneumoniae* or *P. aeruginosa*) [44,47]. No correlation was found between the increase in the mutation frequency and the two concentrations of ciprofloxacin used. The three highest increases in mutation frequency (12-fold, 10-fold, and 6-fold) occurred at both ¼ MIC (strains *P.m.* Bk100 and *P.v.* Sm95) and ½ MIC (strains *P.v.* Sm95 and *P.m.* Dk32) of ciprofloxacin.

Antibiotic-induced mutagenesis as a consequence of environmental stress is most often linked to the induction of SOS response and activation of error-prone DNA repair systems [9,48]. It has been clearly demonstrated that the SOS regulon can be activated when antibiotics are present at much lower concentrations than those used in clinic to eradicate microorganisms [28,49]. Although this effect of SICs of quinolones, especially ciprofloxacin, has been confirmed on various, in particular pathogenic, microorganisms [34,50,51], there are no data regarding *Proteus* bacilli. Hence, after demonstrating the increased mutation rate, we checked whether the applied conditions affected the activation of the SOS regulon genes in the tested microorganisms and could therefore induce this kind of mutagenesis. For this purpose, the mRNA expression level of *racA*—one of the key genes for the activation of this regulon—was investigated.

**Figure 2:**
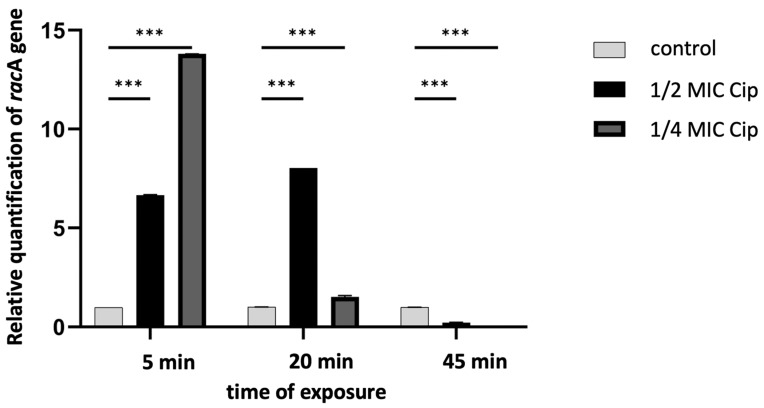
Effect of subinhibitory concentrations of ciprofloxacin on the *recA* gene expression in *P. vulgaris* Sm95 cells. Gene expression levels are represented as log2-fold change relative to the control condition. Asterisk indicates the statistical significance as determined using ANOVA followed by Tukey’s test (*** *p* < 0.001).

Using qRT-PCR and one representative strain, *P. vulgaris* Sm95, which exhibited the highest increase in mutation frequency, we demonstrated that CIP at subinhibitory concentrations (½ or ¼ MIC) induced a rapid and substantial overexpression of the *recA* gene in *Proteus* cells, compared to untreated cells (Figure 2). Significantly, the *recA* expression was upregulated within just five minutes of antibiotic exposure, indicating that the SOS response in *Proteus* bacilli is induced remarkably quickly.

We have hereby demonstrated that the elevated mutation rate in *Proteus* spp. cells may be related to the classic bacterial stress-response pathway, i.e., activation of the SOS response. This type of response, triggered by antibiotic mediated activation of *recA*, results in the activation of error-prone DNA repair systems. Translesion DNA synthesis (TLS) polymerases, especially those lacking intrinsic proofreading activity error-prone Y family DNA polymerase V activated this way, can introduce numerous untargeted changes in the genetic material [8,34,35,49]. Such changes are reflected in differences in the phenotype of the bacteria, including changes towards greater virulence, which from a clinical point of view may have very serious consequences for infected patients.

Undoubtedly, one of the most clinically dangerous and best documented consequences of the low-dose antibiotic-induced mutagenesis is, activated this way, emergence of drug resistance [52,53,54], observed, e.g., in *E. coli*, *P. aeruginosa*, or *Staphylococcus aureus* cells. One of the antimicrobials most frequently described in this context were fluoroquinolones, but this phenomenon was also observed for β-lactams or aminoglycosides [18,55,56].

Bacteria from the genus *Proteus* are opportunistic pathogens usually with a low level of virulence [57]; however, in the case of certain risk groups (e.g., immunocompromised or urology departments patients) the frequency of isolation of these bacilli is much higher. In complicated UTIs, especially when catheterization is performed, *P. mirabilis* is the third most commonly isolated species after *E. coli* and *Klebsiella pneumoniae* [58,59]. Combating the infections caused by *Proteus* spp. is difficult due their recurrent nature and the emergence of an increasing number of resistant (even multi-drug-resistant) isolates of these bacteria [60,61,62]. Although the resistance of *Proteus* spp. has been extensively studied and recently well described [57], these data are mostly limited to increased resistance at high antibiotic concentrations (≥MIC). However, it is not clear whether and how *Proteus* bacilli are affected by sub-MICs of antibiotics, especially during long-term exposure.

In this study, the effects of a 5-day pre-exposure to two SICs (¼ and ½ MIC) of ciprofloxacin—commonly used for *Proteus* infections treatment—on antibiotic susceptibility profiles of the selected clinical *Proteus* spp. strains were examined. First, the MIC values of CIP were established for each strain (Table 1). CIP exhibited the high activity towards the majority of the investigated clinical strains; however, one strain (marked in Table 1 with *) was found to be resistant (according to the breakpoints established by the CLSI). This confirms the still relatively high effectiveness of fluoroquinolones against *Proteus* spp. rods [57]. The resistant strain was selected for the further studies to check whether a high baseline resistance to ciprofloxacin affects changes in drugs susceptibility or not.

Then, the 5-day cultures of all clinical strains, in the presence of CIP (¼ and ½ MIC) and in its absence, were performed and, in consequence, appropriate (“¼ MIC” and “½ MIC“) laboratory variants of all tested strains were obtained; 77.7% of these variants (obtained after the application of low doses of ciprofloxacin) showed changes in the susceptibility to this antibiotic. In most cases, both kinds of variants (“¼ MIC” and “½ MIC“) showed an increase in MIC values (data bolded in Table 1) but in the case of two strains (*P.v.* Bm78 and *P.m.* Dr64), the profile of the obtained changes was much more varied. The “¼ MIC” variants of these strains were characterized by a decrease in the MIC value, with a simultaneous increase in this value for the “½ MIC“ variant of the *P.v.* Bm78 strain. The ratio of changes in the MIC values in most cases ranged from two- to eight-fold but the results obtained for one of the *P. vulgaris* strains (Sm95) were much more spectacular. After exposure of this strain to ciprofloxacin at concentrations below MIC, susceptibility of this strain to the used antibiotic changed dramatically—the noted increase in the MIC values, both for the “¼ MIC” and “½ MIC” variants, ranged from 512- to even 2051-fold. The increase in the resistance of bacteria after long-time pre-exposure to fluoroquinolones (half of the MIC), especially CIP, had been reported earlier for example for *S. pneumoniae* strains. In the case of ciprofloxacin, the decrease in the susceptibility of these microorganisms was associated with an increased efflux, connected with increased expression of *patA* and *patB* genes [63]. In *P. mirabilis* bacteria, molecular mechanisms of resistance to quinolone aside from mutations in DNA gyrase and topoisomerase IV genes (considered as one of the main mechanisms of resistance to this group of antibiotics in a variety of Gram-negative species) also include overexpression of efflux pumps. However, in the case of sublethal treatment, the role of antioxidant defenses has been raised, too [64]. Such a progressive increase in the values of inhibiting concentrations of quinolones, during subinhibitory concentration exposure, is very dangerous in the clinical aspect because the accepted breakpoints of MICs can creep, which may prevent the success of treatment [19,65]. Recurrent infection should be taken into account here—*Proteus* infections are often of this nature.

When resistance via the mentioned antioxidant systems is taken into account, only enhanced MIC values were observed. Interestingly, in our study, not only an increase in the resistance but also a decrease in MIC values was noted—for the “¼ MIC” variants of *P.m.* Dr64 and *P.v.* Bm78 strains (in Table 1 marked in grey). This result was not entirely unexpected since, e.g., for the *P. aeruginosa* PAO1 strain, such a trend had been reported earlier [19]. Interestingly, in the aforementioned studies on *P. aeruginosa*, only an increase in sensitivity to applied antibiotics was observed after pre-exposure to subinhibitory ciprofloxacin concentrations.

The obtained results showed that SICs of ciprofloxacin can influence the antibiotic susceptibility of clinical *Proteus* spp. strains. To ensure that observed changes are the result of stress caused by low doses of CIP, laboratory variants cultivated 5 days without ciprofloxacin were also analyzed. The results for these variants were not appreciably different from those obtained for clinical strains—MIC values in both cases were identical (own research, unpublished). This confirmed clearly that reported changes in drug resistance of *Proteus* spp. strains were the result of the impact of SICs of ciprofloxacin.

Since there is some evidence that SICs of one antibiotic can lead (activating SOS response) to resistance not only to the same class of drugs but also to cross-resistance to quite different antimicrobials [18,31], we decided to test whether the described changes also apply to *Proteus* bacilli. Therefore, we compared the values of MIC of selected antibiotics (in most cases recommended for the investigated bacteria treatment) for clinical *Proteus* strains as well as their “¼ MIC” and “½ MIC” variants.

This study showed that all tested strains underwent changes in the drug susceptibility profiles of the applied antibiotics, to a lesser or greater extent (Table 2). The profile of changes observed for the “¼ MIC” or “½ MIC” variants and their corresponding clinical strains was diverse both in terms of the MIC values and the class of antibiotics used. This variability was observed even in the sensitivity of strains to an antibiotic of the same class as CIP used in the experiment, i.e., norfloxacin (NOR)—both of which are second-generation fluoroquinolones (compare the results in Table 1 and Table 2). The “¼ MIC” and “½ MIC” variants of the three tested strains (*P.m.* 9Bm; *P.v.* Sm95 and Kr83) were characterized, under the tested conditions, by an increase in the MIC values of both CIP and NOR, but the remaining strains did not show such a convergence. Variants of two other strains (*P.m.* Br19 and Dk32) showed a decrease in sensitivity (an increase in MIC values) to CIP, with no changes in sensitivity to NOR (both variants of *P.m.* Dk32 and the “½ MIC” variant of the *P.m.* Br19) and an increase in MIC values to the latter antibiotic (the “¼ MIC” variant of *P.m.* Br19). In the case of the other three strains (*P.v.* Bm78; *P.m.* Dr64 and Dk45*)*, an even more varied profile of changes was recorded. Of all the used strains, only one (*P.m.* Bk100) showed no differences in the sensitivity of both variants of this strain either to CIP or NOR. It should be noted, however, that this was an initially ciprofloxacin-resistant strain. The obtained results are therefore in line with previous reports on the possibility of increasing resistance of bacterial strains to fluoroquinones after exposure to SICs of CIP [31,56,64,66]. Taking into account that antibiotics of this class are often used to eradicate *Proteus* spp. infections, it is necessary to increase awareness of the threat resulting from the possibility of selecting variants with changed sensitivity to these preparations. In particular, the specific feature of these infections—the formation of urinary stones due to the activity of *Proteus* rods—significantly promotes the formation of such niches in the urinary tract where the concentrations of antibiotics considered lethal are not achieved. It was shown that the selection of *Proteus* spp. variants with different susceptibility to fluoroquinolones might result in therapy failure during recurrent infection, even while using an antibiotic from the same group, other than those used during the previous episode.

The results of the presented studies also clearly showed that SICs of ciprofloxacin may induce the development of cross-resistance of *Proteus* strains towards other classes of antibiotics (Table 2). Variants of six of the tested strains (67%) showed changes in sensitivity to gentamicin (GEN), a glycoside antibiotic, after preexposure to SICs of CIP. The vast majority of these changes consisted in a decrease in the sensitivity of the variants to GEN, which was reflected in an increase in MIC values. The level of these changes ranged on average from 2- to 100-fold, but for variants of the two tested strains (*P.v.* Sm95 and *P.m.* Dk32), it was expressly much higher (256–512-fold). In contrast, a 4–8-fold decrease in MIC values of GEN after CIP treatment was observed for both variants of the *P.m.* 9Bm strain. However, no changes in sensitivity to this aminoglycoside antibiotic were noted for variants of the three isolates (*P.v*. Bm78 and Kr83; *P.m.* Bk100). Previous reports had shown varying results regarding changes in sensitivity to this group of antibiotics after exposure to low doses of fluoroquinolones. Neither Cebrian et al. [67], Jørgensen et al. [56], nor Ching and Zaman [68] reported any changes in susceptibility to aminoglycosides (gentamicin, tobramycin, and kanamycin, respectively) after exposure to subinhibitory concentrations of ciprofloxacin. However, the studies by Kohanski et al. [18] showed a relatively small decrease in susceptibility to kanamycin of mutants developed after exposure to low doses of norfloxacin. In contrast, the level of the increases in gentamicin MIC values shown in our studies was within a very wide range (starting from 2-fold, through 50-fold, up to even 256–512-fold) and undoubtedly indicate the emergence in the *Proteus* bacilli population of a subpopulation of mutants showing cross-resistance to aminoglycosides.

Changes in the susceptibility of *Proteus* spp. variants in relation to clinical strains, following treatment with CIP, were also observed in relation to β-lactam antibiotics. The “¼ MIC” and “½ MIC” variants of all tested strains showed changes in sensitivity to all, or almost all of this class antimicrobials. Variants of as many as 78% of strains showed two types of changes in the MIC values—an increase for some antibiotics but a decrease for others. A divergent type of change for two variants of the same strain was also noted (see the results on AMX sensitivity of the *P.m.* Dr64 strain in Table 2b). Variants of the other two strains (*P.m.* 9Bm and Bk100) showed only a decrease in sensitivity to the used antibiotics. The changes in MIC values observed for the used β-lactams, similarly to the antibiotics discussed above, ranged from 4- to 256-fold. This increase in some cases was enough to cross MIC breakpoints and allowed for classifying these variants as clinically resistant to at least one, two, or (in the most extreme case of the *P. m.* 9Bm strain) to almost all used β-lactams.

β-lactam antibiotics are a group of preparations widely used to combat infections of various etiologies. The possibility of developing cross-resistance to them, after previous exposure to low doses of fluoroquinolones, had been previously reported in both Gram-positive [66] and Gram-negative pathogens [19,56,67,68]. The present work shows that this dangerous phenomenon also applies to opportunistic bacteria of the genus *Proteus*. How serious a problem this can pose is evidenced by the fact that some of the tested strains (e.g., *P.v.* Sm95) were initially sensitive to only one (CFD) or two (AMX and CFD) (e.g., *P.m.* Bk100, Br19) antibiotics out of the six β-lactams used. After exposure to fluoroquinolones, some of their variants became resistant to the abovementioned drugs.

When discussing the results obtained in this work, we cannot ignore those that indicate that under the tested conditions, *Proteus* spp. strains may also show an increase in sensitivity to some antibiotics (marked in gray in Table 2a–c). This direction of changes was mentioned by Kumari and co-workers [19], who considered such results very surprising and requiring further research. However, in the light of recent reports and the results of our studies, it seems that the increase in the level of sensitivity should not be considered a rare and isolated phenomenon. Studies on *Salmonella* Typhimurium have shown that collateral sensitivity to aminoglycosides may develop under the influence of ciprofloxacin and tetracycline [69]. The authors even suggest that it would be possible to re-sensitize bacteria to antibiotics, which could help develop effective strategies to combat the increasing drug resistance. However, it seems that this problem should be approached with extreme care, because in our study, the use of CIP was not observed to significantly sensitize *Proteus* strains to the action of gentamicin (only variants of the *P.m.* 9Bm strain showed such a trend).

It should be noted here that the resistance of *Proteus* spp. variants, arising under the SICs of ciprofloxacin, that appeared in all the above-discussed cases was heteroresistance (the population heterogeneity was manifested by different levels of increase in MIC values). As reported earlier, the appearance of cells with even a small increase in resistance in the population may have far-reaching consequences, even if this increase does not yet qualify the strain to be considered resistant according to CLSI standards. Low-level resistance, which may even be overlooked in the studies, allows for variants to persist in the population and, consequently, increase the pool of cells that, on the one hand, are the cause of therapy failure, and on the other hand, may undergo further mutations [3,31,68].

Numerous reports on the growing drug resistance among *Proteus* strains mostly refer to studies taking into account the therapeutic (high-level) concentrations of antibiotics [59,70,71,72,73]. However, the results presented above clearly indicate that, in the case of the discussed microorganisms, SICs of ciprofloxacin may also induce drug resistance, including cross-resistance, and, very importantly, different-level multidrug resistance as well. Reduced or limited drug penetration to the porous structure of urinary stones, as well as an incorrect therapy in *Proteus*-mediated infections, may favor the exposure of these bacilli to sublethal doses of antibiotics. This, in turn, may promote the emergence of variants insensitive to different antibiotics (hence a possible recurrence of infections). The results obtained in this study, together with the knowledge about the frequency of use of fluoroquinolones in the treatment of *Proteus* spp. infections, should be a premise for a very rational and well-thought-out therapy in order to avoid antibiotic stress-induced mutagenesis, leading to drug resistance and thus to treatment failure.

In conclusion, our studies revealed that in *Proteus* spp. clinical strains, long-time exposure to subinhibitory concentrations of ciprofloxacin can lead to overexpression of the *recA* gene and SOS response. Thus, the increased genetic variability, through the enhancement in mutation rate, contributes to the development of resistance, including cross-resistance. This underscores the importance of appropriate antibiotic use to minimize the risk of fostering adaptive traits in bacterial populations. Further studies are needed to elucidate how subinhibitory concentrations of antibiotics influence the broader spectrum of *Proteus* bacterial properties, particularly their virulence factors.

## 3. Materials and Methods

### 3.1. Bacterial Strains

Nine *Proteus* spp. clinical strains used in this study and reported earlier [21] were obtained from urine, wounds, and feces of patients in the Lodz region (Poland) and were stored in Luria broth with 25% glycerol at −80 °C. All strains were kindly provided by D. Drzewiecka, Ph.D., Faculty of the Biology of Bacteria, University of Lodz and are listed in Table 3.

### 3.2. Assessment of the Antibiotics Susceptibility

The drug susceptibility of the investigated clinical strains and their “¼” and “½ MIC” laboratory variants, obtained during 5-day exposure to low doses of ciprofloxacin, was determined in Mueller–Hinton broth by a microdilution method, performed according to the Clinical and Laboratory Standards Institute (CLSI) recommendation [74]. The minimal inhibitory concentrations (MICs) of all used antimicrobials were determined as the lowest concentrations that completely inhibited visible growth. MIC values for the clinical strains were established in triplicate. In the case of “¼” and “½ MIC” laboratory variants, at least ten single colonies of each variant were used to obtain broth cultures and perform susceptibility tests. The following antimicrobials were used in this study: fluoroquinolones: ciprofloxacin—CIP and norfloxacin—NOR (Sigma-Aldrich, St. Louis, MO, USA); aminoglycoside: gentamicin—GEN (Sigma-Aldrich, St. Louis, MO, USA); β-lactam antibiotics: ampicillin—AMP (Fluka, Sigma-Aldrich, St. Louis, MO, USA), amoxicillin and clavulanic acid—AMX (GlaxoSmithKline, London, UK), cephalothin—CFL, ceftazidime—CFD (Bioton, Ozarow Mazowiecki, Poland), cefuroxime—CFX, and cefazolin—CFZ (Sigma-Aldrich, St. Louis, MO, USA).

### 3.3. Culture Conditions for Laboratory Variants Obtaining

A single colony of each clinical strain was inoculated into Mueller–Hinton broth—MHB (BTL, Lodz, Poland). After 24 h incubation at 37 °C, bacterial suspensions were adjusted to the density corresponding to 10^5^ colony-forming units (CFU)/mL. Ciprofloxacin, at the ¼ and ½ MIC values determined for particular strain was added to the cultures, and the bacteria were then exposed to these low doses of the antibiotic over 5 days, at 37 °C with aeration. After every 24 h, broth suspensions were centrifuged, and bacterial pellets were resuspended in the new portion of the Mueller–Hinton medium containing ¼ or ½ MIC of ciprofloxacin. Finally, the cultures were centrifuged, washed twice with fresh medium without the antibiotic, and “¼ MIC” and “½ MIC” laboratory variants of clinical strains were stored with 25% glycerol at −80 °C. Next, 5-day cultures in the MHB without ciprofloxacin were performed in the same way.

### 3.4. Determination of Mutation Frequency

The Rif^S^ to Rif^R^ forward mutagenesis assay was used to determine the effect of SIC of ciprofloxacin on mutation rates. Briefly, overnight cultures of investigated *Proteus* spp. strains were diluted into MHB to an optical density OD_600_ = 0.5. Then, the same amount of suspension (10 µL) with the density described above was added to 5 mL of fresh medium containing ciprofloxacin at subinhibitory concentrations (¼ or ½ MIC) or without the antibiotic. After 24 h at 37 °C, 100 µL of serial dilutions of the cultures was placed on Mueller–Hinton agar supplemented with rifampin (100 µg/mL) and without the antibiotic and the colonies were counted after 24 and 48 h at 37 °C. Mutation frequency was defined as the ratio of CFU count of rifampicin-resistant bacteria to total CFU on antibiotic-free Mueller–Hinton agar. The experiment was performed in tenfold replicates for each clinical and variants strain.

### 3.5. RNA Isolation and Quantitative Polymerase Chain (qPCR) Analysis

The expression of the *recA* gene was analyzed by RT-PCR and quantified relatively in relation to the *rpoD* and *rpoB* reference genes in *Proteus vulgaris* (GenBank, Assembly: GCA_002591155.1 Complete Genome). Ciprofloxacin, a fluoroquinolone antibiotic, was tested as a potential inducer in *Proteus* spp. cultures that had been previously grown for 18 h at 37 °C. The inducer was applied at concentrations equivalent to ¼ and ½ of MIC and samples for RNA isolation were taken at 0 min and after subsequent 20 and 45 min of exposure to ciprofloxacin. A drug-free culture served as the control. Total RNA was extracted using the “Total RNA Mini” kit, and residual DNA was removed with the “Clean-Up RNA Concentrator” (both sets from A&A Biotechnology, Gdansk, Poland). cDNA synthesis was conducted using 1 μg of DNA-free total RNA, RevertAid reverse transcriptase (Thermo Scientific, Waltham, MA, USA), and Random Hexamer Primers (5′-NNNNNN-3′; N = G, A, T, or C) (Thermo Scientific, Waltham, MA, USA) according to the manufacturer’s protocol. Quantitative PCR (qPCR) was performed on a Rotor-Gene Q System (Qiagen, Hilden, Germany) following the previously described protocol, using SsoAdvanced Universal SYBR Green Supermix (2×) (Bio-Rad, Hercules, CA, USA) and gene-specific primers. The reaction conditions included an initial denaturation at 95 °C for 1 min, followed by 40 cycles of denaturation at 95 °C for 20 s, annealing at 60.5 °C for 20 s, and extension at 72 °C for 15 s. A melting curve analysis was conducted by increasing the temperature from 72 °C to 95 °C to confirm amplicon specificity. Relative gene expression levels were calculated using the 2−ΔΔCT method with *rpoD* and *rpoB* as the internal control, following MIQE (Minimum Information for Publication of Quantitative Real-Time PCR Experiments) guidelines [75].

### 3.6. Statistical Analysis

Statistical analysis of the results was performed using Statistica 13. PL software [76]. One-way analyses of variance (ANOVA) and Tukey (HSD) post-hoc test were used to assess the statistical significance of differences in mutation frequency of strains. A *p*-value < 0.05 was considered significant.

## Figures and Tables

**Table 1:** Ciprofloxacin susceptibility of the clinical *Proteus* spp. strains and their “¼” and “½ MIC” laboratory variants.

No.	Strain	MIC of Ciprofloxacin (µg mL^−1^)		
		Clinical Strain	Laboratory Variant	
			“¼ MIC”	“½ MIC”
**1**	*P.m.* 9Bm	0.0078	**0.0312–0.125**	**0.0312–0.0625**
**2**	*P.v.* Bm78	0.0312	0.0039–0.0078	**0.0625–0.125**
**3**	*P.v.* Sm95	0.0156	**8–16**	**8–32**
**4**	*P.m.* Br19	0.0078	**0.0312–0.0625**	**0.0312–0.0625**
**5**	*P.m.* Dr64	0.0625	0.0039–0.0078	0.0625
**6**	*P.v.* Kr83	0.0312	**0.125–0.025**	**0.125–0.5**
**7**	*P.m.* Dk32	0.0078	**0.0156–0.0312**	**0.0156–0.0312**
**8**	*P.m.* Dk45	0.0156	0.0156	**0.0625–0.125**
**9**	*P.m.* Bk100 *	2	2	2

“*P.m*.”—*Proteus mirabilis*; “*P.v*.”—*Proteus vulgaris*. Strain exhibiting resistance to ciprofloxacin is marked with *. Data showing increase in MIC values are bolded. Data showing decrease in MIC value are marked in grey.

**Table 2:** Susceptibility profiles of investigated strains after SICs of ciprofloxacin treatment. Result as shown as MIC values (µg ml^−1^). (**a**) Results obtained for urine isolates. (**b**) Results obtained for wound isolates. (**c**) Results obtained for feces isolates.

(**a**)									
**Strain and Their** **½ and ¼ MIC Laboratory Variants**		**ANTIBIOTIC**							
		***Penicillin***		***Cephalosporin***				***Aminoglycoside***	***Quinolone***
		**AMP**	**AMX**	**CFL**	**CFX**	**CFZ**	**CFD**	**GEN**	**NOR**
***P.m.*** **9Bm**	Clinical	1	1	4	1	4	1	2	1
	½ MIC	**256–512**	**128–512**	**256–512**	**256–512**	**256**	1	0.5–0.25	**8–16**
	¼ MIC	**256–512**	**8–16**	**256–512**	**128–512**	**128**	1	0.5–0.25	**8–32**
***P.v.*** **Sm95**	Clinical	128	16	64	32	512	0.015	1	0.031
	½ MIC	**>512**	16	**512**	**256–512**	512	**1–2**	**256–512**	**32–64**
	¼ MIC	**>512**	2–4	**512**	**512**	512	**0.125–0.25**	**128–512**	**16–32**
***P.v.*** **Bm78**	Clinical	32	4	32	128	512	0.031	0.25	0.015
	½ MIC	**256–512**	4	**128–256**	16–32	512	0.031	0.25	**0.062**
	¼ MIC	**128–256**	4	**64–128**	16–32	64	**0.125–0.25**	0.25	**0.062**
(**b**)									
**Strain and Their** **½ and ¼ MIC Laboratory Variants**		**ANTIBIOTIC**							
		***Penicillin***		***Cephalosporin***				***Aminoglycoside***	***Quinolone***
		**AMP**	**AMX**	**CFL**	**CFX**	**CFZ**	**CFD**	**GEN**	**NOR**
***P.m.*** **Br19**	Clinical	16	4	8	0.031	8	0.062	0.01	0.062
	½ MIC	16	4	8	**0.5–2**	8	**0.25–1**	**0.5–1**	0.062
	¼ MIC	0.5–2	0.5–1	0.5–2	**0.125–1**	8	**0.25–0.5**	**0.5–1**	**0.25–0.5**
***P.m.*** **Dr64**	Clinical	1	1	0.5	0.031	4	0.031	1	1
	½ MIC	1	0.125–0.5	0.5	**0.5–1**	0.5–1	0.031	**2–4**	0.015
	¼ MIC	1	**2–4**	**2–4**	**1–2**	4	**0.062–0.25**	**4–8**	0.015–0.031
***P.v.*** **Kr83**	Clinical	64	2	16	8	16	2	2	0.062
	½ MIC	64	**32–128**	**256–512**	**64–128**	**256–512**	0.031–0.062	2	**8–16**
	¼ MIC	**128–512**	**16–128**	**256–512**	**256–512**	**512**	0.015–0.062	2	**0.5–1**
(**c**)									
**Strain and Their** **½ and ¼ MIC Laboratory Variants**		**ANTIBIOTIC**							
		***Penicillin***		***Cephalosporin***				***Aminoglycoside***	***Quinolone***
		**AMP**	**AMX**	**CFL**	**CFX**	**CFZ**	**CFD**	**GEN**	**NOR**
***P.m.*** **Dk32**	Clinical	512	128	512	128	256	0.062	2	0.125
	½ MIC	512	128	512	128	256	0.015–0.032	**512**	0.125
	¼ MIC	512	4–8	512	128	256	0.062	**>512**	0.125
***P.m.*** **Dk45**	Clinical	0.5	0.5	0.062	0.015	0.125	0.015	0.5	0.062
	½ MIC	0.062–0.125	0.125–0.25	**0.5–1**	**0.062–0.125**	**0.25–0.5**	0.015	**4–8**	0.062
	¼ MIC	0.062–0.125	0.062–0.125	**0.5–1**	**0.062–0.125**	**0.5–1**	**0.031–0.062**	**2–8**	**1–2**
***P.m.*** **Bk100**	Clinical	32	4	64	16	16	0.031	512	8
	½ MIC	**64–128**	**16–32**	64	16	**32–64**	**4–8**	512	8
	¼ MIC	**128–256**	4	64	16	16	0.031	512	8

The following abbreviations were used: NOR: norfloxacin; GEN: gentamicin; AMP: ampicillin; AMX: amoxicillin and clavulanic acid; CFL: cephalothin; CFD: ceftazidime; CFX: cefuroxime; CFZ: cefazolin. Data showing increase in MIC values are bolded. Data showing decrease in MIC value are marked in grey.

**Table 3:** *Proteus* strains used in this study.

No.	Strain Species	Strain Name	Source of Isolation
1	*P. mirabilis*	9Bm	Urine
2	*P. vulgaris*	Bm78	Urine
3	*P. vulgaris*	Sm95	Urine
4	*P. mirabilis*	Br19	Wound
5	*P. mirabilis*	Dr64	Wound
6	*P. vulgaris*	Kr83	Wound
7	*P. mirabilis*	Dk32	Feces
8	*P. mirabilis*	Dk45	Feces
9	*P. mirabilis*	Bk100	Feces

## Data Availability

The original contributions presented in this study are included in the article. Further inquiries can be directed to the corresponding author.
